# A study of the required sustainability-driven institutional and behavioural mechanisms to tackle the anticipated implications of agricultural water price shocks: a system dynamics approach

**DOI:** 10.1038/s41598-023-42778-8

**Published:** 2023-09-16

**Authors:** Marzieh Momeni, Vahid Razavi, Sina Zahedi, Farshad Momeni, Kourosh Behzadian, Neda Dolatabadi

**Affiliations:** 1 Faculty of Engineering, School of Civil Engineering, University of Tehran, Tehran, Iran; 2 Research Institute for Science, Technology and Industry Policy, Sharif University of Technology, Tehran, Iran; 3 Department of Civil Engineering, Monash University, Clayton, VIC 3800 Australia; 4 Faculty of Economics, Allameh Tabataba’i University, Tehran, Iran; 5 School of Computing and Engineering, University of West London, London, W5 5RF UK

**Keywords:** Environmental social sciences, Environmental economics, Psychology and behaviour, Socioeconomic scenarios, Sustainability

## Abstract

Economic policies for managing agricultural water use are often complicated by the challenge of using water prices as an efficient economic tool when other non-economic concerns are involved in the decision-making process. This study aims to analyse the impact of water pricing policies on preserving agricultural water resources in Iran. This study applies a system dynamics approach to simulate the system performance and behaviour of stakeholders and the economic implications. Our finding shows that water pricing policies will likely fail due to low water price elasticity and if there are lack of institutional and physical infrastructure, alternative professions, manufacturing technology, education, and training opportunities. The results also illustrate how agricultural water price increase (AWPI) fails to reduce water consumption in the absence of an adequate institutional arrangement. Also, it shows how the lack of advanced institutional infrastructure in the presence of physical infrastructure enhances pervasive overuse and destructive competition among stakeholders by increasing the area under cultivation. In the discussion, the paper portrays a way out of the decision-making body by following AWPI effects on water conservation in the agricultural sector as the most significant water consumer. It investigates the absence and subsequent presence of specific institutional conditions and evaluates training and enhancing farmers' skills and alternative career source with higher income and technology as the architecture of good environmental governance. Finally, it concludes that a series of inclusive measures must be considered to increase the elasticity of the water price. These measures must stimulate farmers towards pursuing the goals of global sustainable development and enhancing social welfare.

## Introduction

Water price is one of the essential decision-making drivers in managing water consumption in the agricultural sector^1^. In many countries, agriculture is the leading consumer of water; hence, water price is critical in the sector. For example, Iran's conventional irrigation practices account for nearly 90% of the country's total water consumption^2^. Therefore, Iran's Sixth Development Plan has taken immediate steps to raise the country's agricultural water price^3^. The most prominent policy reasons for increasing agricultural water tariffs to regulate agricultural water use are low irrigation efficiency, low crop production, and depletion of groundwater resources^4^. However, this action will likely engender public opposition to increased water prices. Due to mismanagement and conflicting interests among stakeholders involved in the water system, insufficient efforts have been made to unify the management of water resources. Madani et al. identify three significant causes for the current water crisis in Iran^5^: (1) high population growth and its disproportionate distribution. (2) Inefficiency of the agricultural sector; and (3) mismanagement of water resources and a thirst for development. All of these have resulted in increased water demand, severe water shortages, decreased groundwater levels, deteriorating water quality, and increased ecosystem losses^6–8^. In addition, the lack of laws for contemporary irrigated farms leads to an increase in under-cultivated land rather than a reduction in water usage^9^. Some decision-makers concluded that water tariff adjustment could be a powerful economic leverage to overcome the long-term challenges of water supply services, i.e.,^10,11^. In contrast, independent studies indicated that the usefulness of water price adjustment is ambiguous. Enforcing water price adjustment before preparing relevant institutional infrastructure can be evidence of unprincipled confrontation in dealing with this issue, i.e.,^12,13^. Such policies can induce considerable changes in the subsistence of those relying on agricultural water subsidies. Hence, the agricultural water pricing policy may result in ambiguous and unexpected effects on farmers’ subsistence^14^. The nature of these types of studies needs to model feedback mechanisms among different drivers. A System Dynamics (SD) approach is suitable for analysing behavioural aspects of stakeholders and consumers under different scenarios before implementing their policies in place^15,16^. Therefore, we investigate SD modelling based on the assumptions of future executive plans for Iran's development. This paper aims to construct an SD model to analyse how farmers in a developing country like Iran react to rising water prices. Our study aims to follow and implement appropriate environmental policies and prerequisites for increasing agricultural water prices in the context of Iranian conditions as a developing country.

## Methodology

In this paper, a system dynamics (SD) model has been developed to examine the socio-economic responses of farmers to rising water price tariffs under plausible scenarios. The SD approach has been chosen due to its ability to incorporate the key components of water resource management, socio-economic factors, institutional strategies, and technical limitations. These strategies and limitations can include policies related to water price adjustments, mismanagement in water resources, farmers' behavioral patterns, living conditions, and their interactions with other national policies. Farmers may show different socio-economic responses when dealing with these policies. One method of obtaining these results is to conduct a direct questionnaire for farmers to fill out. An anonymous questionnaire filled out by 100 farmers confirmed the hypothesis of a clear association between rising water prices and decreased water use. In addition, the authenticity of the farmers' responses was reviewed by a committee of 25 experts in the field. Experts were also given anonymous questionnaires with questions that addressed a variety of variables and ideas in order to conduct a professional analysis of the topic of water price increases. The answer to each question is set as (1) a list of multiple alternatives divided into four categories: good, moderate, poor, and extremely poor/none; and (2) a list of numerous options divided into four categories: good, moderate, poor, and very poor/none. (3) Yes or No response; (4) many alternatives for the respondent to choose from or write; and (5) a mix of the preceding formats. This questionnaire was created using the research's synthesized model, which included theoretical frameworks of institutional and behavioral economics.

### Model development

**Figure 1:**
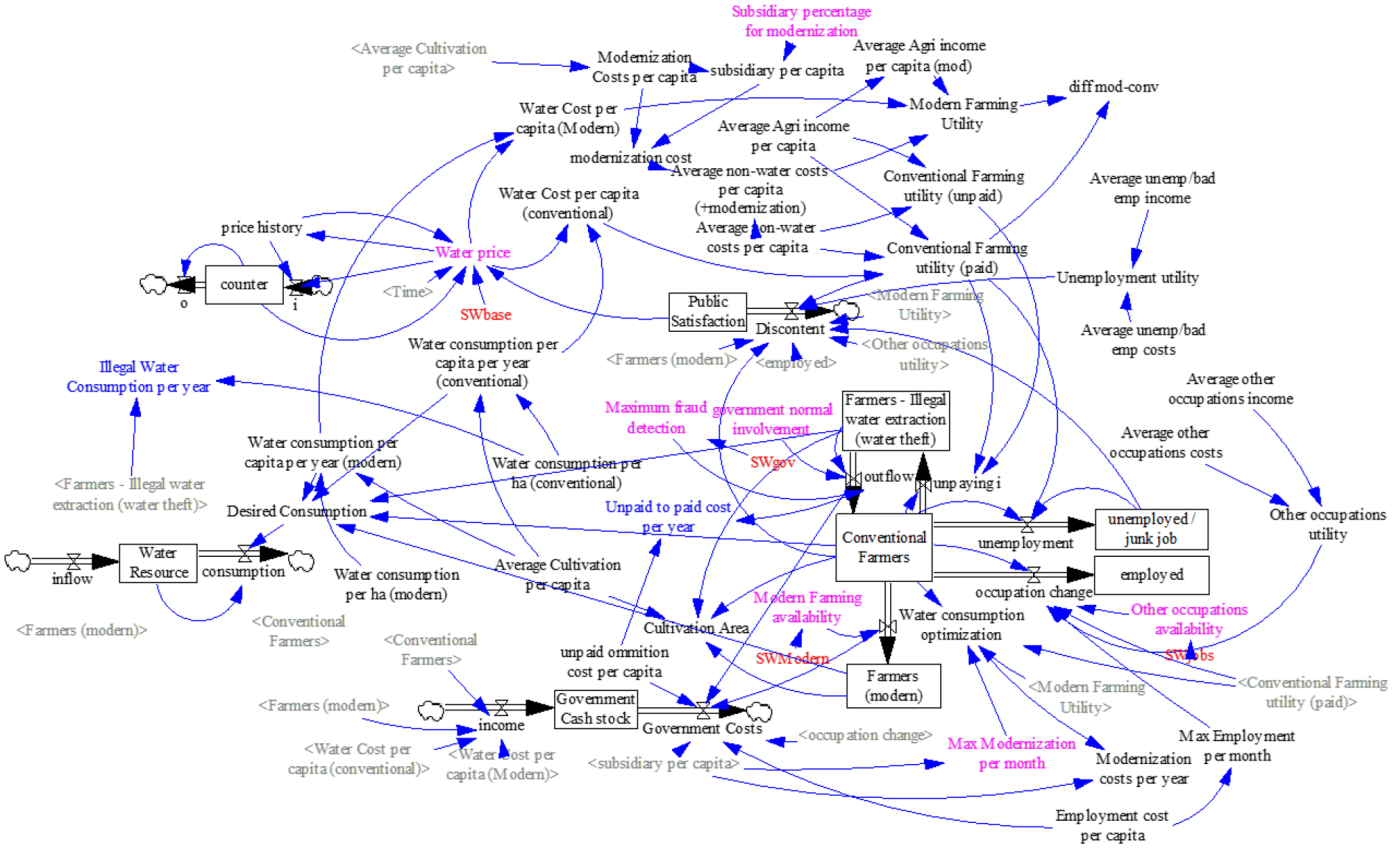
The schematic SD model.

The SD model developed here simulates the impacts of increasing water prices through various dynamic elements in water systems. Figure 1 illustrates the background of the model, feedback mechanisms, and the elements developed in this study.

### Farmers and change of occupation

The SD model is developed based on the farmers’ preferences collected reflected by their answers to the questionnaire. The following five options were offered to the farmers in the face of the increase in agricultural water price:

- Continuing conventional agriculture (i.e. business as usual)
- Continuing conventional agriculture as well as turning to illegal water extraction (e.g., illegally drilled water extraction wells)
- Replacing conventional irrigation methods with modern and sustainable alternatives
- Abandon farming and taking casual/junk jobs or opting for unemployment
- Abandon farming and taking other occupations

These five options constitute the main stock variables in the SD model. All individuals in each variable (which is an indicator of farmers’ livelihood) would decide whether to change their occupations based on utility variables associated with different occupations. The utility of each occupation is estimated based on monthly benefit and cost relevant to fundamental variables such as the input costs and final product prices. The SD model considers the effects of the decline in the utility of occupation and waning social satisfaction on individuals. The social satisfaction variable is a function of the utility of the individual’s occupation and is used as an indicator of social discontent. Variations in social satisfaction may trigger public pressure on the authorities to cancel the implemented policies.

The SD model also includes the decisions made by policymakers that tend to impact the utility and availability of each subsistence. The following major elements influenced by policy-makers are defined in the SD model:

- Irrigation water price
- Availability of modern and sustainable irrigation (availability of governmental subsidies and equipment)
- The ratio of irrigation modernization costs covered by governmental subsidies
- Availability of alternative occupations: This variable includes access to the required infrastructure to change livelihood such as education, publicity, providing conditions for development and prosperity of alternative occupations
- The level of governmental control on illegal water extraction (water theft)

The SD model also considers a cap for some operational and enforcement measures such as detecting illegal water extraction (maximum water theft detection), allocating budget for upgrading irrigation systems (maximum modernization per month), and allocating budget for new occupations (maximum employment per month).

### Key performance indicators

The analyzing policies are evaluated in the developed model through key performance indicators which include total cultivation area, the number of farmers (traditional farmers, farmers shifting to modern and sustainable irrigation, and farmers tending to illegal water extraction), volume of agricultural water consumption (total agricultural water consumption and illegal water extraction or water theft per year), amount of budget allocated by the authorities (irrigation modernization subsidy, detection of illegal water extraction budget for agricultural use, cost of creating alternative occupations), the number of farmers that shift from farming to other occupations (casual/junk jobs and alternative jobs), and public satisfaction during the implementation of the increase in irrigation water price.

### Model dynamics

Two types of model dynamics are defined here that are influenced by the system variables under the policies defined in the scenarios. 

#### Public satisfaction dynamics

Increasing water price can result in high costs of conventional agricultural products, however, such increase can lead to the low profit margin without a proportional increase in the price of product in the market. This can have negative implications for the agricultural industry and public satisfaction level. The negative consequences can be further exacerbated if neither modern irrigation nor alternative occupation is available for farmers, inevitably forcing them to either continue agriculture with low profit or choose unemployment. Under such circumstances, a decline in social satisfaction and more pressure on responsible authorities to revert water price to its initial value is expected. This can happen when pressure exceeds the threshold of public satisfaction function.

#### Occupation change dynamics

If the factors affecting the utility of an occupation change, an individual with that occupation may switch to occupations with higher utility in the market. This can culminate in fluctuating water consumption and fixed governmental costs. Moreover, occupation change would alter the social satisfaction and discontentment, which may in turn trigger the "public satisfaction and pressure on the authorities" dynamics discussed above.

The Supplementary material includes the complete questionnaire and model assumptions.

### Scenarios

In this paper, a base scenario is assumed in which farmers continue their traditional farming practices and no policy is implemented by the government. We developed four scenarios based on the materials discussed in the methodology and the suggestions made by consultants' experts about the agricultural water price increase (AWPI):

- Scenario #1: AWPI without supporting policies.
- Scenario #2: AWPI with governmental attempts to limit illegal water extraction (LIWE).
- Scenario #3: AWPI-LIWE and facilitating irrigation modernization (FIM).
- Scenario #4: AWPI-LIWE-FIM and the transition to alternative occupations with higher income and technology.

**Figure 2:**
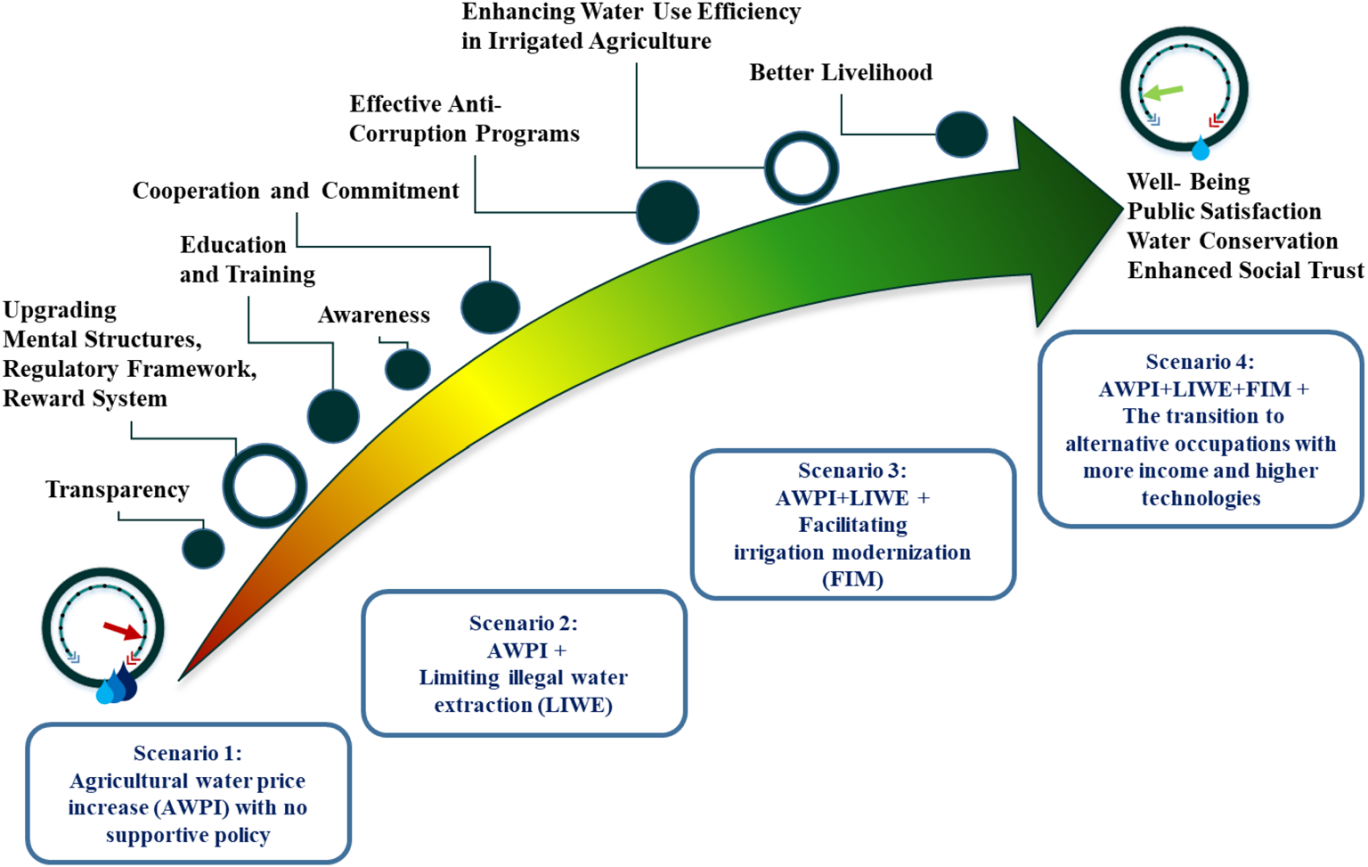
Four scenarios developed for our study and the corresponding required measures.

These four scenarios indicate the conditions and challenges of the farming community affected by the policy of agricultural water increase in the 100 monthly time steps. Table 1 gives the details of the model setup for these scenarios. Also shown in Fig. 2 are the different scenarios alongside measures that should be taken to guarantee the effectiveness of pricing policies. It is noteworthy that institutional and behavioural economics frameworks were employed in this study. The simulated fluctuations are a consequence of the government’s decisions to either increase the price of water or revert the decision. These decisions are made upon exceeding a pre-determined level of dissatisfaction. Increasing the price of water or canceling this decision is the only variable with step-wise variations which results in fluctuation of other variables.

**Table 1:** The similarities and differences between scenarios set up inthe model.

Model set-up information	Scenario #1	Scenario #2	Scenario #3	Scenario #4
Starting time step of AWPI policy	Time step 13	Time step 13	Time step 13	Time step 13
Possibility of withdrawal of adopted AWPI policy	Available	Available	Available	Available
The threshold for public satisfaction functions to stop the AWPI policy based on the number of traditional farmers	Below the 33%	Below the 33%	Below the 33%	Below the 33%
The threshold for public satisfaction functions to restart the AWPI policy based on the number of traditional farmers	Equal or above the 33%	Equal or above the 33%	Equal or above the 33%	Equal or above the 33%
Utility index for conventional farming (traditional farming)	Available	Available	Available	Available
Utility index for conventional farming with illegal water extraction (traditional farming with water theft)	Available	Available	Available	Available
Utility index for farming with irrigation modernized equipment	Available	Available	Available	Available
Utility index for casual/junk jobs and unemployment	Available	Available	Available	Available
The number of conventional farmers	✔	✔	✔	✔
The number of conventional farmers tending to illegal water extraction	✔	✔	✔	✔
The number of conventional farmers shifting to casual/junk jobs and unemployment	✔	✔	✔	✔
The number of conventional farmers shifting to alternative occupations	×	×	×	✔
Illegal water extraction (water theft)	Moderate restricted	Severely restricted	Severely restricted	Severely restricted
Budget for irrigation modernization	Low	Low	High	High
Budget for transition to alternative occupations	Not-considered	Not-considered	Not-considered	Determined

### Data

‏ The data used to develop the model were based on the Statistical Centre of water resources management database in Iran^17^ for the water year 2016–17. Various researchers acknowledge that a combination of drought, mismanagement, and lack of equipment are likely to be responsible for‏ ‏the water crisis in Iran^18,19^. Table 2 presents the assumptions relevant to ‏the agricultural development section of Iran's Sixth Five-Year Economic Development Plan. Most information in Table 2, except the suggestion to consider a training budget for alternative occupations, was taken from Iran's Sixth Development Plan. Also, there is a "Supplementary material" section to explain the conditions of Iran’s agricultural sector.

**Table 2:** Suggestions set into the different scenarios of the model.

The variables of the case study	Status quo	Suggestions set out in the model
Irrigation water price (USD per m^3^)	0.0034	0.0188
Budget for reducing illegal water extraction (reducing water theft) (USD per year)	18,750,000	56,250,000
Budget for irrigation modernization (USD per year)	81,250,000	468,750,000
Budget for training alternative occupations (USD per year)	–	468,750,000
The average cost of training each person (USD per capita)	–	937.5

### Ethics approval and consent to participate

The study was conducted according to the guidelines of the Declaration of Helsinki and approved by the ethics committee of the economic planning and development department of the faculty of Economics, Allameh Tabataba’i University.

### Informed consent

Informed consent was obtained from all individual participants included in the study.

## Results

**Figure 3:**
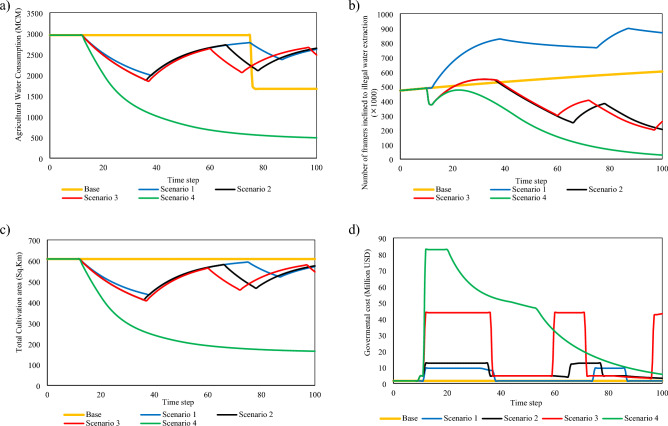
The outcomes of significant variables for four scenarios developed by the model: (**a**) water consumption in agriculture, (**b**) the number of farmers inclined to illegal water extraction, (**c**) total cultivation area, (**d**) total governmental cost to achieve planned aims.

Focusing on water consumption indicates that agricultural water use would be reduced only in Scenario #4 by providing alternative occupations for farmers. In Base Scenario, with the variables (including water price) remaining constant and the inclination of traditional farmers towards holding on to their primary jobs, water use will continue to remain high, depleting resources and decreasing their availability. The dynamics involved in dealing with the diminishing water resources are complex; however, investigating these dynamics is not in the scope of this paper (Fig. 3a). Different scenarios of policy-making discussed in this paper may cause variations in unpaid and illegal water withdrawal. As presented in Fig. 3b, the highest tendency to extract illegal water is observed in Scenario #1 due to a lack of possible alternatives for farmers in response to the water price increase. The total number of farmers attempting illegal water extraction decreases sharply in Scenario #2 and Scenario #3 of policy-making as the authorities allocate afinancial budget; however, as discussed before, none of the first three policies leads to a decrease in water consumption. In Scenario #4 of policy-making, the authorities could shift the farmers to occupations with higher utility with which agriculture cannot compete, even if agricultural water price decreases.

The diagram of the cultivated area under four states of policy-making has similar behaviour to water consumption. In the first three scenarios, the cultivation area remains between 5 and 6 million hectares (land area under wheat cultivation) as farmers continue their conventional occupation. As a result of providing alternative occupations by the government, a considerable percentage of farmers changed jobs, and the cultivated area decreased. Figure 3c can be rehtruf analysed from the perspective of reduction in cultivated area and its different adverse effects. In Base Scenario, As the number of traditional farmers does not change, the area under cultivation will remain constant. Considering all four scenarios and comparing all scenarios from the very first perspective, one can suggest that scenario #4 requires a more generous budget compared to Scenarios #1, #2, and #3. In Scenarios #1 and #2, the governmental costs fluctuate as the result of changes in irrigation water price so that there are significant changes for the time steps with high water price (0.0188 USD per cubic meter) compared to those with low water price (0.0034 USD per cubic meter). The only difference between Scenarios #1 and #2 is the difference in the budget allocated for resisting illegal water extraction. As shown in Scenario #3, the efforts are not adequate and would have serious adverse financial effects on farmers in the long run. Under scenario #3, farmers can only achieve suitable utility by advancing modernization in the agricultural field. There is no way for them to free themselves from this occupation. Besides, this substantial investment would not reduce water consumption on the planning horizon. Therefore, scenario #3 has the same fate as the first two. In Scenario #4, the initial cost is considerably higher than other scenarios due to facilitating the transition to alternative jobs and the spread of irrigation modernization. This scenario involves reduction in the governmental costs during the planning horizon due to the decline in the number of farmers who attempt illegal water extraction, a rise in the completed modernized farming projects, and a decrease in the number of farmers who shift to alternative jobs (Fig. 3d).

**Figure 4:**
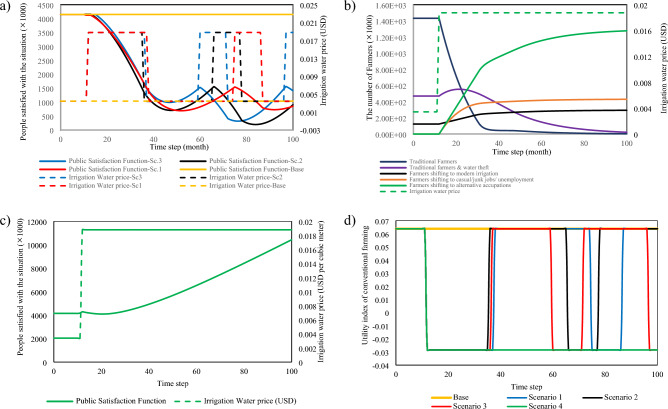
(**a**) Public satisfaction difference between Scenarios 1, 2, and 3, (**b**) the feedback environment between the number of farmers and water price adjustment, (**c**) public satisfaction during policy implementation for scenario 4, and (**d**) utility index comparison between all four scenarios for conventional farming.

Figure 4a shows that in the first three scenarios, the model considers the situation in which agricultural water prices increase so that farmers will be forced to bear the burden of training and equipment costs on their own due to a lack of institutional and physical infrastructures. According to scenario #1 and scenario #2, if farmers cannot pay the imposed costs, their general satisfaction will decline, forcing the government to reverse its policies. In scenario #2, applying punishments when no incentives exist to prevent the worsening of the consequences or when the cost of avoiding the penalty is too high would only impair public satisfaction and lead to the policy's eventual failure. Regarding scenario #3, this sector does not show significant improvements even though loans are given to aid agriculture's modernization. Because the agricultural sector relies heavily on water resources, offering incentives to improve water efficiency does not represent a viable alternative to imposing regulations for water price adjustment. This is because the price of water directly affects farmers' subsidies. As a result, it is necessary to limit the number of people who rely on agriculture for their subsistence by introducing new productive employment. Reducing the number of farmers is possible by providing free and accessible educational and training infrastructure and creating jobs to replace farming. Through the educational training infrastructure, people seeking new occupations will be taught skills related to production and modern agriculture (Fig. 4b).

The difference between scenario #3 and scenario #4 comes from the financial allocation for training for alternative jobs. This shifting to alternative jobs causes many workers to have different alternatives for their livelihoods, and the public satisfaction function shows a distinct behaviour compared to previous scenarios. Farmers will have confidence in the government's policy if it provides institutional infrastructure such as crop insurance and loans to farmers who want to employ advancing agricultural technology, as well as physical infrastructures such as water delivery networks and irrigation channels^20,21^. Once other farmers learn about the benefits of risk-taking farmers who moved towards agricultural modernization while accepting the increase in water prices and the significant profits gained through the government's agricultural water regulation method, they will be attracted to this approach. Farmers who do not own land will also benefit from the government's support and education and training initiatives. These initiatives will assist them in gaining specialized skills that will enable them to work in other areas. As a result, the public satisfaction function will be on the rise, and the government will meet the objectives outlined in the water price increase policy (Fig. 4c)^22,23^. The policy of water price adjustment continues without resetting to the previous price, and irrigation modernization would continue until the last time step, even though the utility index for traditional farming is negative (Fig. 4d).

The government's investment cost in the agricultural sector in Scenario #4 is 53,17.7, and 2 times greater than in Scenarios #1, #2, and #3, respectively. The policy of increasing water prices in Scenario #4 is successful due to this difference in the initial investment inthe agricultural sector. The government pushes farmers to change their farming techniques and equipment by increasing the water price. However, they understand farmers’ basics for their livelihoods by funding the training of traditional farmers for modern agriculture or alternative jobs. The policies would likely fail in other scenarios since conventional agriculture is no longer profitable and modern agriculture requires expert technicians. However, there are no educated technicians and fewer farm labourers. The policies would fail when the public satisfaction function decreases below the threshold of 33% of agricultural society supporting the policy. These results show that loan payments and pushing farmers to modern farming are necessary but not enough to pursue the agricultural water price increase policy. If policymakers are willing to continue the agricultural water price increase policy, training and alternative occupations for farm laborers must be considered.

## Discussion

The interdependence of agriculture and subsistence is deemed a vital issue in developing countries.

Thus, the model begins when policymakers implement the agriculture sector's strategy to increase water prices. In the model, stakeholders and effective actors interact to influence each other. The feedback mechanism of SD simulates the interaction between all players to find the results of each scenario. In this model, formal institutions include water pricing regulations, laws supporting farmers with loans and incentives to modernize irrigation equipment, training programs, educational infrastructure, and incentives to reduce water consumption^24^.

The model employs these institutions to define four scenarios to lay the background for interaction between formal and informal institutions.

It is suggested that farmers are more inclined to trust conventional agriculture despite its environmental shortcomings. Owing to the limitations in securing farmers’ livelihoods, the sensitivity of farmers to variations in the initial cost of agriculture, including water price, is relatively high. In addition, poverty, discrimination, cognitive limitations, and a lack of access to the infrastructure required for modern and technological production have alienated these people from education, resulting in a lack of alternative jobs in case farming no longer maintains its utility. The lack of alternative jobs and training for other occupations has persuaded farm workers to continue farming even with low utility. The integration of knowledge limitations and the absence of technical training does not permit them to implement modern farming or quit farming. This may explain why farmers do not prefer the approach that brings them more profit, even if there is effective instruction about sustainable water-based agriculture.

Furthermore, being solely a part of the agricultural labour force and not owning a piece of land as their property has made many farmers dependent on their limited income. Therefore, they are vulnerable to a slight change in water price. It is necessary to facilitate institutional and physical infrastructure towards a speedy transfer to alternative occupations and modernization of tools for a more water-saving production in advance of decisions on the possible water price increase and their consequent social unrest. Thus, economic tools should be leveraged as the last step to preserve natural resources and prevent the likely social turmoil.

To this end, the most vital requirement is establishing a suitable stimulus system and taking measures to prevent discrimination among farmers and other members of society from further aggravation. This can be attributed to unproductive activities like casual or junk jobs being rewarded and a rise in the utility of unemployment compared to farming. The combination of such factors will likely cause a country to fall into a vicious circle of food security disruptions, water resources loss, and intensifying environmental chaos.

Upon initiation of implementing a water price increase policy without procuring the required physical and institutional infrastructures, policies aimed at preserving common-pool resources, providing food security, and conservation of the environment will be neutralized^25,26^. These policies, considered a sign of nowlessness in policymaking, will impose devastating effects on the environment and society.

An example of a chaotic situation is a repeated implementation of the water price increase policy in a specific period and attributing its expected failure to inappropriate execution and diminishing public satisfaction, as demonstrated by the results of Scenarios #1, #2, and #3. It is likely that such measures will accelerate the destruction of the environment and promote destructive competition in the exploitation of natural resources, intensifying the tragedy of the commons^25,27^. In the face of such disruptions, we have devised the 4th scenario, which involves preventing farmers from wasting more resources to increase production to secure their livelihoods and directing them towards raising efficiency instead. The utopia envisioned in Scenario #4 can never materialize until all the significant contributors of a developing country to environmental policymaking agree to a comprehensive environmental protection program.

Unjustified inequalities, in addition to short-sighted and unsustainable signalling, are deemed as two catastrophic repercussions of the sudden increase in the price of key products according to a 44-year-long experience obtained from implementing such adjustment policies of Iran. In developing economies and from a sustainability standpoint, appreciation of the real sequences is the most vital issue concerning pricing policies^28^. The necessity of adjusting prices is indisputable; however, it must be noted that such a decision must be the last link in a chain of institutional and physical infrastructure-related measures toward sensible and revitalizing decision-making^29^. Thus, a far-sighted and sustainability-driven decision-making body should primarily be concerned with putting on its agenda sufficient alternatives for improving farmers’ livelihood, which will also warrant food security and environmental protection in addition to modernization of production through arranging physical and institutional arrangements^30^.

The price change is subsequently considered if necessary. A wise policy-maker should know that inflation is the root cause of instabilities and disruptions in people’s individual and collective livelihoods. This issue is comprehended through a thorough assessment of counter-sustainability and inequality-propagating impacts of inflation. In a nutshell, should there be a comprehensive evaluation of policy sequences, the economy will turn into a win–win game. Otherwise, the price increase will become a priority of decision-makers, leading to devastating threats to human lives, the environment, and national-level development.

**Figure 5:**
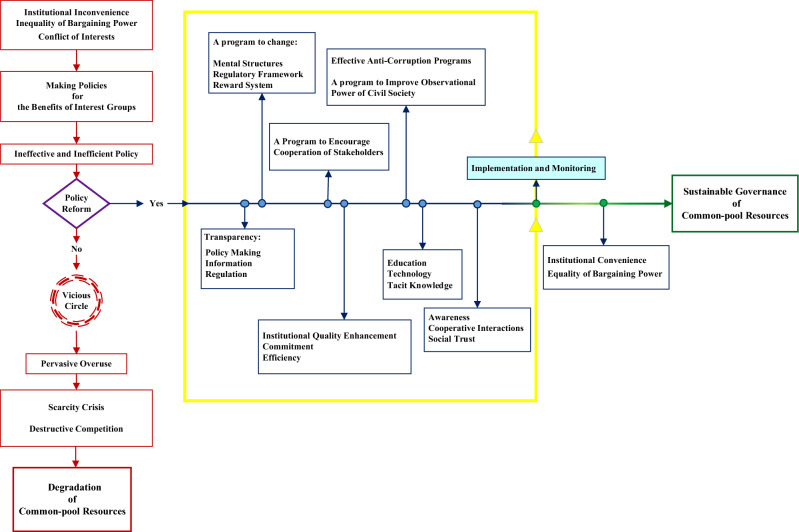
The schematic of institutional reform for an implemented policy.

Figure 5 depicts the real-world policymaking process for reaching an ecologically sustainable outcome. According to Fig. 5, cooperation and coordination among various governmental bodies, effective actors, and stakeholders can result in a long-term outcome if conflicts of interest between stakeholders and effective actors are resolved during the various coordination meetings. Only when there is a balance of power between decision-makers, stakeholders, and effective actors can an all-encompassing logic in resource conservation be realized^31^. As a result, the power imbalance in the system can lead to an intended-unsustainable consequence. The construction of a transparent, responsive, and democratic decision-making organization can be implemented in a demanding society as the way out of this scenario^32^. For a sustainable water use policy, it is necessary to adjust formal and informal institutions and the financial budget. Before any modification in the ultimate price of agricultural water, the decision-making body must address all flaws and give equal opportunities to all effective actors and stakeholders^33^.

## Conclusion

This paper presented the effects of the agricultural water price increase policy using a system dynamics model under four different scenarios. In each scenario, different reactions were observed according to the policy. None of the first three scenarios had a sustainable impact on decreasing water consumption. However, Scenario #4 was the only one capable of creating proper dynamics for farmers' transition from traditional practices to modern methods and alternative jobs. The outcome of Scenario #4 highlights the role of job utility in improving farmers' satisfaction in the face of the price change scenario. It illustrates that investment in training modern farming and creating new jobs for these societies is a way out of the tragedy of the commons. Executing this approach requires free public education and support incentives to be provided by the government.

The following can be noted in this paper:

1. Given Iran's current circumstances, increasing farmers' costs will complicate their welfare conditions and lower their utility function. Implementing such a policy in the current economic conditions of a developing country will increase farmers' production costs and decrease their income. This will motivate them to seek alternative careers. Since these farmers have no other production skills, their only option is to immigrate for a new career. This could be as simple as becoming casual labourers or taking up casual/junk jobs such as vendors, and this could be a strong motivation for illegal water extraction.
2. Limiting and restricting access to vital common-pool resources will decrease public satisfaction among those whose subsidence depends on it. Therefore, it is necessary to consider opportunities for career changes.
3. An increase in water prices can increase the popularity of modern agriculture. Such circumstances will naturally result in the decline of traditional agriculture as many of the farmers who own their lands will hire fewer workers due to the application of smart and electronic equipment. They are also more likely to have employees whose skills are helpful for modern agriculture. As a result, many traditional farmers will lose their jobs and need to learn additional skills related to alternative jobs or modern agriculture. Thus, the price change policy will only be successful if the government provides training for alternative jobs and modern agriculture skills at no cost, along with incentives for using the latest irrigation equipment.
4. A smart decision-making structure must be advised that inequality-inducing consequences of price increase policies will disrupt any economic, social, and ecological balance and result in an escalation of environmental destruction, immigration, robbery, divorce, juvenile delinquency, dwelling in slums, and higher crime rates in lieu of establishing cooperation toward sustainable development.
5. The government and other decision-making bodies are seriously responsible for addressing issues caused by sudden price increases; Not only because they are the main consumers and thereby the most vulnerable to inflation in an under-developed economy, but also due to the possibility of them being captured because of the undermined legitimacy and financial instability.
6. A Lack of access to water equals the demise of humans, just like other species. It must be noted that water is dissimilar to a large group of other goods in that reduction of its consumption through a price increase cannot be selected as the goal of a policy.
7. A thorough appreciation of sequences and acknowledging that adjusting the price of key products must be the final link in a chain of measures taken by a sustainability-driven government rather than the initial one. This is the true essence of methodological evaluations, revealing the pragmatic consequences of shock therapy.

### Supplementary Information

Supplementary Information.

## Data Availability

The datasets used and/or analysed during the current study available from the corresponding author on reasonable request.
