# Supplementary material for: A study of the required sustainability-driven institutional and behavioural mechanisms to tackle the anticipated implications of agricultural water price shocks: a system dynamics approach
Source: Sci Rep. 2023 Sep 16;13:15397. doi: 10.1038/s41598-023-42778-8 (PMC10505165; doi:10.1038/s41598-023-42778-8)
Supplement: Supplementary file 1 — Supplementary Information. [file 41598_2023_42778_MOESM1_ESM.docx]

**Supplementary**

# The questionnaire

The questionnaire (Table 1) described in the methodology is as follows:

Table 1: The questionnaire designed to develop the model

| **No.** | **Question** | **Type** | **Farmers’ viewpoints** | **Experts’ viewpoints** |
| --- | --- | --- | --- | --- |
| 1 | How much is the knowledge of the responder/farmer about ways of saving water in the agricultural sector?  (Experts answered this question based on the data extracted from farmers’ solutions regarding ways to reduce water consumption) | Ordinal | 18 good answers  33 moderate answers  35 poor answers  14 very poor/none answers | 0 good answers  3 moderate answers  5 poor answers  17 very poor/none answers |
| 2 | In case of the water price increase, does the responder/ farmer deem the adopted policy unfair? | Binary | 100 Yes answers  0 No answers | 25 Yes answers  0 No answers |
| 3 | In case of endangering the livelihood of farmers due to the water price increase, does the responder/ farmer feel threatened by the circumstances? | Binary | 65 Yes answers  35 No answers | 25 Yes answers  0 No answers |
| 4 | In case of the water price increase, does the responder/farmer identify water theft as a way of securing his/her needs? | Categorical | 29 Yes answers  71 No answers | 25 Yes answers  0 No answers |
|  | If the answer to the above question is yes, will the responder/farmer suggest others do that too? |  | 9 Yes answers  20 No answers | 4 Yes answers  21 No answers |
| 5 | If the water efficiency is improved without changing its price, will the responder/farmer keep the area under cultivation unchanged? | Binary | 42 Yes answers  58 No answers | 0 Yes answers  25 No answers |
| 6 | In case of the water price increase and stability of the economic status, will the responder/farmer be inclined towards choosing another career in addition to farming? | Categorical | 81 Yes answers  19 No answers | 18 Yes answers  7 No answers |
|  | Farmers: if the answer to the above question is yes, does the responder have any skills that could be used in production or service other than farming?  Experts: if the answer to the above question is yes, considering the farmers’ living conditions and the available facilities, do farmers have the opportunity to learn skills other than traditional farming? |  | 21 Yes answers  60 No answers | 3 Yes answers  15 No answers |
| 7 | In case of free modern farming training courses are offered by the government for the responder/ farmer, will you/he/she attend these courses? | Categorical | 90 Yes answers  10 No answers | 19 Yes answers  6 No answers |
|  | If the answer by the responder/farmer is ‎yes, how modern agriculture ‎training is effective in improving irrigation ‎conditions? |  | 57 good answers  21 moderate answers  13 poor answers  0 very poor/ none answers | 0 good answers  5 moderate answers  8 poor answers  6 very poor/ none answers |
| 8 | In current economic conditions and assuming the water price increase, to which of these options is the farmer more inclined?   1. Immigration and changing to another career 2. Water theft | Optional | 64 chose option one  36 chose option two | 19 chose option one  6 chose option two |
| 9 | Farmers: does the payment of financial aids and low-interest loans motivate the responder/farmer to reduce water consumption in agriculture?  Experts: does the payment of financial aids and low-interest loans for irrigation modernization lead to sustainable water resources operation in agriculture? | Binary | 88 Yes answers  12 No answers | 7 Yes answers  18 No answers |
| 10 | With the water price increase in current conditions and assuming the impossibility of immigration for the responder/farmer, which of these options will he/she prefer?   1. Alternative career 2. Water theft and pursuit of farming | Categorical | 69 chose option one  31 chose option two | 14 chose option one  11 chose option two |
|  | Farmers: if the answer to the above question is option one, which of these options is the closest to your selected career?   1. Simple labourer 2. Turning to skills other than driving 3. Being a salesperson in a shop 4. Being a vendor 5. Real estate agent 6. Being employed in the public or private sector as a guard, for instance 7. Investment in a new job 8. Driving a taxi or as a transportation agent   Experts: if there is no possibility of water theft for farmers, in which areas will most of them get involved?   1. Casual/junk jobs (such as brokers, vendors, etc.) 2. Production-oriented jobs 3. Jobs requiring specific skills 4. Jobs that provide services (such as labourer, salesperson, driver, etc.) |  | 19 chose option one  3 chose option two  7 chose option three  15 chose option four  8 chose option five  1 chose option six  4 chose option seven  12 chose option eight | 9 chose option one  1 chose option two  5 chose option three  10 chose option four |
|  | Farmers: if the responder’s choice is option two, will the aggravating economic conditions, in addition to the growing monitoring measures and penalties imposed for water theft, drive the farmers angry towards the government’s actions?  Experts: if there is no possibility of choosing ‎another career, what effect does increase ‎monitoring agencies and imposing ‎significant penalties for crimes such as water ‎theft have on the farmers’ protest threshold?‎  After imposing severe penalties and increasing government monitoring, the public protest (uprising) threshold will:   1. Decrease 2. Remain the same 3. increase |  | 26 Yes answers  5 No answers | 17 chose option one  6 chose option two  2 chose option three |
| 11 | Farmers: in case of the water price increase and ‎the government’s inability to supply subsistence ‎for farmers, will the responder voice his/her ‎objection to his/her conditions and ‎the government’s negligence?‎  Experts: in case of the water price increase in current conditions, will the increasing public discontent following the price increase lead to exceeding the farmers’ protest threshold? | Binary | 61 Yes answers  39 No answers | 21 Yes answers  4 No answers |
| 12 | Farmers: does the government care about the farmers’ protests regarding their livelihood?  Experts: will the continuing protests of farmers in response to the water price increase lead to a halt and failure in implementing the policy?   1. Yes 2. No 3. Depends on the government’s actions in managing the circumstances | Binary / Optional | 13 Yes answers  87 No answers | 6 chose option one  6 chose option two  13 chose option three |
| 13 | In the case of modernizing farming and the decline in traditional farming practices, which of these options is most beneficial for their new career?   1. Training for alternative careers 2. Training skills related to modern agriculture 3. Options one and two | Optional | 19 chose option one  17 chose option two  64 chose option three | 2 chose option one  5 chose option two  18 chose option three |
| 14 | Farmers: considering the climate of developing countries and in the case of modernizing agriculture, which of these critical elements of irrigated/ water-dependent farming is farmers’ most significant concern?   1. water resources 2. fertile soil 3. weather conditions 4. taxes and tolls 5. seeds 6. pests 7. Selling agricultural products 8. Repair and maintenance of agricultural equipment 9. Type of fertilizer and management of its distribution 10. Labourers and skilled workforce   Experts: according to farmers’ response and after ‎imposing limitations on the water as the ‎most vital resource required for irrigated farming, will the general ‎satisfaction level of the farmers revert to ‎the previous level (before the price ‎increase) in the face of the government’s ‎measures such as training alternative ‎farming skills and allocating budget for ‎modernization of farming?‎ | Optional / Binary | 61 chose option one  15 chose option two  9 chose option three  8 chose option four  1 chose option five  0 chose option six  4 chose option seven  0 chose option eight  0 chose option nine  2 chose option ten | 1 Yes answer  24 No answers |
| 15 | Farmers: as far as the responder is concerned, in case of the water price increase, which measures will increase farmers’ satisfaction with the government’s actions?   1. Payment of financial aids and subsidies to farmers 2. Training careers related to modern agriculture or alternative jobs   Experts: which of these options do you consider ‎effective in terms of increasing public satisfaction?   1. Continue to pursue policies of the water price increase and training skills related to alternative careers along with modern agriculture 2. Continue to pursue policies of water price increase in addition to payment of subsidies and unemployment insurance to the farmers in need 3. Reverting the price to the initial value and intensifying the monitoring measures, imposing maximum penalties on over-consumers and those who commit acts of water theft 4. Reverting the price to the initial value to prevent the decline in public satisfaction | Optional | 76 chose option one  24 chose option two | 17 chose option one  5 chose option two  3 chose option three  0 chose option four |

# ‎ **Model assumptions**

It is supposed to ‎assess the effects of the water price changes on the utility of farming and the feasibility of ‎alternative occupations for farmers, affecting agricultural water consumption. Inthe ‎case of increasing social pressure to more than a specific threshold, the government ‎needs to adjust the price or return it to its initial level. It is also assumed that the authorities here have two choices for adjusting agricultural water price: (1) increasing ‎ to real prices or (2) decreasing or remaining the existing price by allocating agricultural subsidy. The following assumptions are also considered in the SD model:

- The transition ratio between two occupations is dependent on the ratio of ‎utility of those two occupations.
- The utility of occupation is considered to be equal to the ‎ratio of income on the cost of that livelihood (Other factors like occupational ‎adaptation, occupation simplicity etc. are neglected).
- A threshold is considered for the rate of ‎changing from traditional to modern irrigation for agricultural lands (about 3 hectares per month).
- Wheat is assumed to be the principal agricultural product as the main agricultural crop for Iran. Hence, modern ‎irrigation would lead to 43 percent reduction in water demand and 10 percent growth in crop yield.
- The authorities would consider budget‎ to confront illegal water ‎withdrawals (e.g. illegal water wells).
- Regarding the public satisfaction function, the model would continue based on assumed water price or return to its previous price by decreasing the threshold of public satisfaction.
- 12 monthly time steps are required for agricultural water price change.

# Scenarios:

## Scenario 1: AWPI without supporting policies

In this scenario, there is no support from policy makers after imposing the water price shock. The following observations can be noted from the results of scenario 1.

### Occupations’ ‎ utility differences ‎

By increasing water ‎price, the utility of farming using conventional and modern irrigation diminishes. This depletion is more ‎considerable in conventional irrigation due to more water consumption. Considering ‎illegal water extraction, water price change does not influence traditional agriculture. ‎Due to low water consumption in non-agricultural occupations, they are assumed to be negligible in this study and hence not modeled. Prior to water price revision, the utility of traditional ‎agriculture was better than modern one. After ‎changing the water price, the ‎utility of both kinds of agriculture decreases due to an increase in ‎their costs. The ‎noteworthy point is that after increasing water price, modern agriculture has ‎better utility ‎than the conventional ones due to less water use and ‎more crop yields in modern agriculture. However, due to the absence of any budget to limit illegal water extraction, the utility of conventional farming with illegal water extraction would remain constant at the same level for all 100 monthly time steps. Thus, most conventional farmers would be inclined to continue traditional methods of irrigation, but with illegal water extraction (water theft increase).

### ***Occupation change dynamics*‎**

After imposing price shock, the population of conventional farmers is indirectly proportional to the water price. This is due to of the lack of incentives for guiding farmers to alternative occupations and also the ‎limitation of financial aids and infrastructures to transform conventional farms to modern and sustainable ‎ones and hence conventional farmers are mostly employed in casual/junk jobs, or started illegal water extraction from water resources. As ‎there is no support from the authorities in this scenario, modern agriculture ‎follows a slight ascending pattern.‎ An increase in casual/junk jobs and unemployment as well as the decline in ‎conventional ‎agriculture utility leads to an increase in discontent resulting in undoing ‎the ‎price revision policy (decreasing water price to its initial amount) discussed in the ‎next ‎dynamic.‎

### **Public satisfaction and the effect of public pressure on water price ‎increase policy**

Due to the decline in agriculture utility, farmers’ unemployment, ‎approaching ‎casual/jobs, and public discontent is raised that eventually would ‎lead to an increase in public ‎pressure on the authorities. Therefore, the ‎authorities have to reset the water price to its ‎initial value due to pressures of ‎occupations with low financial benefits (or sometimes with ‎financial loss).‎ As a result of the water price reset, some of the conventional farmers would return to their ‎occupation and agricultural water consumption would return to the critical limit. Besides, a delay between the process of returning water price to its previous price and the effectiveness of this policy on public satisfaction is set out in the model to illustrate the nature of gradual satisfaction and dissatisfaction regarding the imposed policies. When the number of satisfied people with an imposed policy drops to below 33% -37% of the total population, the water price returns to its previous price. By increasing the price of irrigation water in the Scenario 1, the number of farmers shifting towards illegal water extraction increases at each time step of implementing the new price. Thus, a slight rise can be seen in the number of farmers prone to illegal water extraction. This shifting is affected by the value of irrigation water in the planning horizon.

## Scenario 2: AWPI with governmental attempts to limit illegal water extraction

In this scenario, the authorities increase preventive actions for reducing illegal water extraction. It is assumed that the authorities try to decrease water ‎consumption using ‎preventive policies and tripled theft prevention budget.

### ***Occupations’ ‎ utility differences* ‎**

In this scenario, the behavior of occupations utility is as same as the previous policy-‎making state, i.e., more decline in conventional agriculture utility than the ‎modern ‎one, and consistency of the other occupations.

### ***Occupation change dynamics*‎**

Because of the increase in theft prevention budget, the amount ‎of ‎illegal water withdrawal decreases so that the number of farmers decreases in the planning horizon; ‎However, since conventional farmers do not have alternative occupations yet (which were ‎less water consumption), they shifted towards casual/junk occupations or ‎unemployment. ‎Meanwhile, because of the low level of allocated financial support to ‎irrigation ‎modernization, few farmers are able to transfer to modern and sustainable agriculture. ‎Therefore, due to ‎unemployment or casual/junk occupations, as well as low level of agriculture ‎utility, public ‎discontent, and pressure increase.‎ As public satisfaction function drops below the protest threshold (the percentage of public satisfaction drops below the level of 33% - 37% among all residents in agricultural communities), the authorities have to reset the water price. Similar to the scenario 1, there is a delay between the policy of returning water price to previous price and the effectiveness of this policy on farmers’ lives. Therefore, the public satisfaction function continues to decrease for two months after turning the prices back. In addition, ‎comparing this state of policy with the first one, a noteworthy point is that ‎public ‎satisfaction is influenced by the conditions of this scenario (illegal water extraction prevention) more considerably than it was in the first scenario and the process of ‎water price changes occurs in smaller time steps compared to the scenario 1. To justify this fact, the conventional ‎farmers, who tend to ‎withdraw water illegally in response to the water price increase, ‎would have to continue their old occupation and eventually approach unemployment or ‎being employed in casual/junk ‎jobs and ultimately join the discontented population.‎ Thus, the process of returning to previous water price can slightly increase the rate of public satisfaction function in scenario 2 compared to scenario 1. By implementing illegal water extraction preventive policy without considering ‎supporting policies (supporting irrigation modernization and less water-consumptive ‎occupations), the outcome is not optimal. Moreover, the outcome would not get any ‎better by allocating more budgets to illegal water extraction prevention actions, as public discontentment ‎and pressure lead to water price reset anyway.

## Scenario 3: AWPI with governmental attempts to limit illegal water extraction and facilitating ‎irrigation modernization

In this state of policy, the authorities follow irrigation modernization strategies, in ‎addition ‎to illegal water extraction preventive actions. In this regard, they offer more financial ‎aids, ‎infrastructures, and equipment for national irrigation modernization.

### ***Occupations’ ‎ utility differences*** ‎

Based on Fig. 5a, because of incentives and subsidies offered for irrigation modernization, ‎the utility of modern and sustainable agriculture is higher than previous simulations (even more than the ‎utility of illegal water extraction). After water price increase, although the utility of ‎modern agriculture decreases to less than illegal water withdrawal, its utility is still ‎considerably more than conventional agriculture. This result illustrates that the third scenario can be effective only when the agricultural water price still remains at its previous level, which is equal to 0.0034 USD per cubic meter.

### ***Occupation change dynamics*‎**

Even though an annual budget is allocated to irrigation modernization, the rate ‎of ‎conversion from conventional to modern and sustainable agriculture is slow due to the high demand ‎for ‎modernization. Hence, the unemployment rate, casual/junk job employment, and illegal water extraction are still high. These factors lead to a rise in social discontent, ‎public pressure, and ‎eventually, force the government to reset the water price. However, we cannot underestimate the fact that since the irrigation water price returns to its previous value, the ‎acceleration of public satisfaction function would increase during the rising periods compared to Scenario 1 and Scenario 2 so ‎that three periods for water price adjustments. This result indicates that the utility of considering facilities for irrigation modernization as well as limited illegal water extraction for conventional farming can propel farmers towards modernizing irrigation systems during the periods when water price is set to return to its previous price.

## Scenario 4: AWPI with governmental attempts to limit illegal water extraction, facilitating ‎irrigation modernization, as well as facilitating the transition to alternative occupations

In this scenario, in addition to water withdrawal fraud prevention and irrigation ‎modernization policies, the authorities provide the basis (conditions) for conventional ‎farmers’ shift towards modern agriculture.

### **Occupations’ ‎ utility differences ‎**

The utility of modernized farming is clearly far better than conventional farming. Therefore, farmers partially shift to model agriculture when experiencing water price shock.‎ Analyzing occupation change dynamics indicates that the fourth scenario of policy-making is ‎able to generate the desirable shift of conventional farmers to alternative occupations.

### **Occupation change dynamics‎**

The occupation utility of conventional farmers, who shift to modern ‎irrigation ‎ increases, contributing to satisfaction of ‎the ‎society and preventing social discontent (leading to water price reset in ‎previous ‎states of policy-making).‎ The rate of farmers’ occupation change to alternative ‎occupations has risen which leads to growing contentment. This shifting to alternative jobs causes many workers to possibly face different alternatives for their livelihoods and the public satisfaction function depicts a different behavior compared to previous scenarios. The policy of water price adjustment thus continues without any reset to the previous price. The utility of conventional farming decreases below 0 due to the stable high irrigation water price as well as presence of alternative occupations so that the total number of farmers decrease compared to previous scenarios and some of them who are eagerly interested in farming such as land owners would attend to the programs of facilitating modernized farming.

## Comparing different stages of policy making based on different indices

### **Agricultural water consumption**

Focusing on water consumption indicates that agricultural water use would be ‎reduced only by providing the basis for farmers to ‎shift to alternative occupations.

### **The number of conventional farmers inclined to illegal water extraction**

Different scenarios of policy-making discussed in this paper may cause variation in ‎unpaid ‎and illegal water withdrawal. The highest tendency to ‎extract illegal water is observed in the first scenario which is due to a lack of possible ‎alternatives for ‎farmers in response to the water price increase. The total number of farmers who attempt to illegal water extraction ‎decreases sharply ‎in second and third scenarios of policy-making as the authorities allocate ‎financial budget; however, as discussed before, none ‎of the first three ‎policies leads to water consumption decrease. In the fourth scenario of ‎policy-making, the ‎authorities would be able to shift the farmers to occupations with ‎higher utility with ‎which agriculture cannot compete, even if agricultural water price ‎decreases.

### **Area under cultivation**

The cultivated area under four different states of policy-making has ‎similar behavior ‎to that of water consumption. In the first three scenarios, the cultivated area ‎remains between 5 and 6 ‎million hectares (area of land under wheat cultivation) as ‎farmers continue their conventional ‎occupation. In the fourth scenario and due to providing ‎alternative occupations by the authorities, a ‎considerable percentage of farmers change ‎their job and cultivated area diminishes. It can be ‎analyzed from the perspective of ‎reduction in cultivated area and its different negative effects ‎which is not the intent of ‎this paper.

### **Total Governmental costs**

Considering all four scenarios and by comparing all scenarios from very first perspective, one can suggest that the scenario 4 requires more generous budget compared to Scenarios 1, 2, and 3. In the first two scenarios, the governmental costs fluctuate as the result of changes in irrigation water price so that there are significant changes for the time steps with high water price (0.0188 USD per cubic meter) compared to those with low water price (0.0034 USD per cubic meter) and the only difference between Scenario 1 and Scenario 2 is the difference of the budget allocated for resisting illegal water extraction. In Scenario 3, the efforts, in the long run, would lead to serious negative financial effects on the implemented policy such as dependence of ‎governmental costs on changes in water price and undergoing a difficult situation due to the significant cost of these efforts, resulting in the lack of considering alternative jobs, the fluctuations of modernized irrigation utility, and moving ‎towards modernization in the farm fields perceived as the only way among farmers to reach ‎adequate utility by continuing farming even accepting the costs of modernization for their livelihoods. Besides, this huge bulk of investment would not reduce water consumption in the planning horizon. In Scenario 4, the initial cost is considerably higher than other scenarios (about 82.8 Million USD per month, 993.6 Million USD for the first year, and total value of 3.46 billion USD for the entire planning horizon) as a result of facilitating transition to alternative jobs as well as the spread of irrigation modernization. The governmental costs reduces during the planning horizon due to the decline in the number of farmers who attempt to illegal water extraction, the rise in the completed modernized farming projects as well as the decline in the number of farmers who shift to the alternative jobs.

# Case Study

The additional information in the case study part is as follows:

Iran is located in the Middle East, surrounded by Persian Gulf and Oman Sea from the south, ‎Turkey and Iraq from the west and Nakhchivan autonomous republic, Armenia, and Republic of ‎Azerbaijan, from the northwest, and Caspian Sea from the north and Turkmenistan from the northeast and Afghanistan and Pakistan from the east, as shown in Figure 1. With an area of 1,648,000 square Kilometers, Iran is ranked as the seventeenth largest country in the world. Except for the western and northern coastal areas, Iran's climate is predominantly arid and semiarid [1,2,3], with rainfall primarily determined by geographical latitude and topographical height [4]. The average annual precipitation in Iran is about 250 mm (Figure 2). The western Mediterranean oscillation's pressure centre has affected most of Iran's precipitation. Due to a lack of precipitation, which resulted in a smaller amount of accessible surface water, groundwater is the primary source of water supply for municipal and agricultural usage, particularly in arid and semi-arid regions [5]. According to the Water Resources Management Company of Iran, the agriculture sector is the largest consumer of water resources in Iran, as seen in Figure 3a. According to the twenty-year average of total water usage, the agriculture sector accounts for 91.15 percent of total water consumption in Iran (this research will challenge these officially-announced statistics). Furthermore, groundwater resources have provided approximately 59 percent of total water use. Figure 3b demonstrates that groundwater has been Iran's primary water supply for household, industrial, and agricultural usage during the last 20 years.


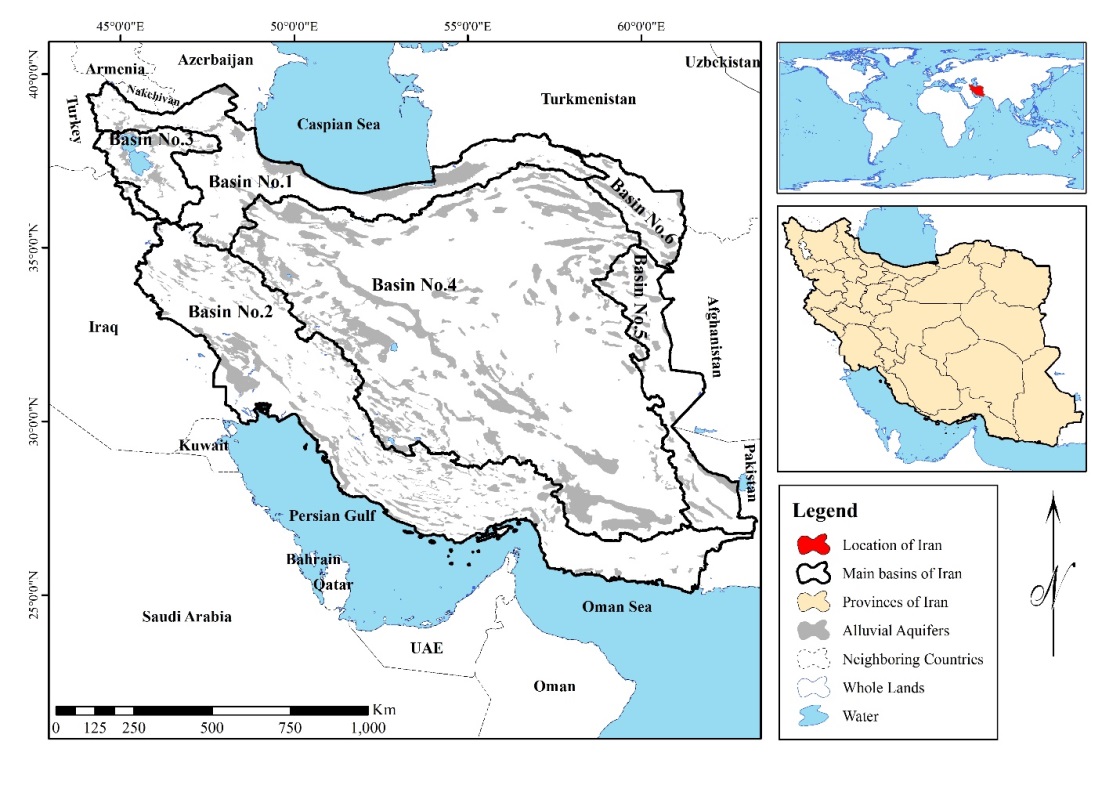


Figure 1: Location of Case Study

Figure 2: Annual precipitation of Iran considering different basins

| a) | b) |
| --- | --- |
|  |  |

Figure 3: Water consumption in different main sectors (a) and water use percentage of available water resources (b)

Because Iran is located in an arid region, just 33 percent of the country's total surface area is suitable for farming. However, due to poor soil quality and a lack of appropriate water resources, large areas of eligible land are not under cultivation. The rest of Iran's farmland is rain-fed, which is dependent on precipitation [6]. Most efforts for the empowerment of farmers and rural communities guided by populist decision makers – such as major subsidization of water and energy to help farmers – have failed and are being cancelled due to a lack of a long-term comprehensive strategy [7]. In the water year 2014-15, the percentage of irrigated areas equipped with irrigation technology is around 25% of the total, as shown in Figure 4. What is clear is that farmers have lost the desire to increase production efficiency due to free and cheap water [8]. Iran's average irrigation efficiency is between 35 and 40%. [9,10]. Figure 3s depicts the total area of irrigated areas equipped with under-pressure technology from 1993-1994 to 2014-15.

Figure 4: The percentage of Irrigated lands equipped with irrigation technologies compared to traditional irrigation technics‎

Farming is one of the most common vocations in Iran, with over 3.5 million people today employed in farmlands. Figure 5 depicts the population percentage in each employment for Iran's major sectors and basins.

Figure 5: Total areas of Irrigated lands equipped with under-pressure technologies

According to Figure 6, the agriculture industry employs 22.55 percent of the entire employment in Iran.

Figure 6: The percentage of labourers/workers‎ in different sectors of Iran

All data were collected from Iran's Statistical Center [11]. Some assumptions based on Iran's regional situation were made to create the model, as shown in Table 2.

Table 2: the data used for model development

| Criteria | Reference crop | Total number of Farmers in irrigated farmlands | Number of farmers in modernized farm fields | Number of farmers in conventional farm fields |
| --- | --- | --- | --- | --- |
| Measure | Wheat | 3913000 | 313037 | 3599963 |
| Criteria | Total cultivation area under irrigation (ha) | Total cultivation area (ha) | The cost of irrigation modernization (USD per ha) | The price of wheat (USD per Kg) |
| Measure | 6132421 | ‎11837908 | 4062.5 | 0.328 |
| Criteria | The percentage of farmers prone to water theft (%) | The annual average income of each family in the agricultural community ‎(USD per year)‎ | The costs for producing crops (USD per Kg) | The annual average income of wheat cultivation from irrigated farm fields (USD per ha) |
| Measure | 23.26 | 4048 | 0.2188 | 1029.57 |
| Criteria | The amount of wheat production in irrigated farmlands (Kg per ha) | Water consumption in modernized farming (m3 per ha) | Water consumption of traditional agriculture (m3 per ha) | The average number of individuals in each family |
| Measure | 3137.7 | 6000 | 3400 | 4 |

In 2016, the price of each cubic meter for farming was 110 Iranian Rials, as reported by the Water Resources Company of Iran However, starting from 2018, the Iranian currency faced significant fluctuations due to economic challenges. In order to tackle this predicament and establish a feasible path for numerous developing nations, we applied the nominal exchange rate of 1 USD to 32,000 Iranian Rials, based on the 2017 rate, for our computations.

Furthermore, for the adjusted water pricing, we referenced the water cost outlined in Iran’s Sixth Five-Year Development Plan (2017–2021), which was set at 600 Iranian Rials (equivalent to around 1000 Iranian rials in 2022). We converted this amount to USD using the same nominal exchange rate index of 1 USD to 32,000 Iranian Rials.

**Reference**

[1] Fallah, B., Sodoudi, S., Russo, E., Kirchner, I., & Cubasch, U. (2017). Towards modeling the regional rainfall changes over Iran due to the climate forcing of the past 6000 years. *Quaternary International*, ***429***, 119–128.

[2] Mansouri Daneshvar, M. R., Ebrahimi, M., & Nejadsoleymani, H. (2019). An overview of climate change in Iran: facts and statistics. *Environmental Systems Research*, ***8***(1), 1–10.

[3] Sodoudi, S., Noorian, A., Geb, M., & Reimer, E. (2010). Daily precipitation forecast of ECMWF verified over Iran. *Theoretical and Applied Climatology*, ***99***(1), 39–51.

[4] Razmi, R., Balyani, S., & Mansouri Daneshvar, M. R. (2017). Geo-statistical modeling of mean annual rainfall over the Iran using ECMWF database. *Spatial Information Research*, *25*(2), 219–227.

[5] Jafary, F., & Bradley, C. (2018). Groundwater irrigation management and the existing challenges from the farmers’ perspective in central Iran. *Land*, ***7***(1), 15.

[6] Mesgaran, M. B., Madani, K., Hashemi, H., & Azadi, P. (2017). Iran’s land suitability for agriculture. *Scientific Reports*, ***7***(1), 7670.

[7] Madani, K. (2016). Editorial.“Water Crisis in Iran: A Desperate Call for Action.” *Tehran Times*.

[8] Nikouei, A., & Ward, F. A. (2013). Pricing irrigation water for drought adaptation in Iran. *Journal of Hydrology*, ***503***, 29–46.

[9] Alizadeh, A., & Keshavarz, A. (2005). Status of agricultural water use in Iran. *Water Conservation, Reuse, and Recycling: Proceedings of an Iranian-American Workshop*, *4*, 94–105. National Academies Press Washington DC, USA.

[10] Nazari, B., Liaghat, A., Akbari, M. R., & Keshavarz, M. (2018). Irrigation water management in Iran: Implications for water use efficiency improvement. *Agricultural Water Management*, *208*, 7–18.

[11] Statistical Center of Iran. Database, https://www.amar.org.ir.
